# Advances in Retrograde Technique for Coronary Chronic Total Occlusions

**DOI:** 10.2174/1573403X1002140506124653

**Published:** 2014-05

**Authors:** Tony J. DeMartini

**Affiliations:** Prairie Cardiovascular Consultants, 401 E. Carpenter, Springfield, Illinois, USA

**Keywords:** Chronic total occlusion, percutaneous coronary intervention, retrograde technique.

## Abstract

Despite a short lag period since its development the retrograde approach has been increasingly integrated within the treatment strategies for the percutaneous treatment of coronary chronic total occlusions. This review article discuss which anatomical features argue most powerfully for its use, the specific skills required for its uptake and the technology which has facilitated these developments.

## INTRODUCTION

The retrograde approach for recanalization of coronary chronic total occlusions (CTO) using collateral channels was first described by Japanese operators [1, 2]. The retrograde approach involves crossing a collateral channel, usually categorized as either epicardial or septal, in order to gain access to the distal cap of the CTO. 

The main reason to go retrograde is the anatomic features that make the antegrade approach less likely to succeed. These anatomic features include an ambiguous proximal cap and the distal cap at a bifurcation. Failure of recanalization of the CTO antegrade and overall procedural efficiency are additional reasons to do the retrograde approach. Lesion length and calcification do not affect the initial approach to the CTO.

Initially, the retrograde approach was complex and reserved for highly skilled operators. Crossing collateral channels with coronary guide wires involves navigating small vessels with tortuosity. If the distal vascular bed of the target vessel was successfully wired in a retrograde fashion, delivering gear, whether balloons or catheters, was difficult. Balloons and microcatheters were not designed to cross such small, tortuous vessels. 

Wiring true lumen to true lumen is one option but this can time consuming and have a low likelihood of success if the occlusion was long, tortuous or calcified. Controlled antegrade retrograde subintimal tracking (CART) was another approach [1]. The procedure of CART involves delivering a balloon from the retrograde side, getting into the subintimal space with both the antegrade and retrograde equipment and then producing a common subintimal space. Overall, this allowed a wire to go from the true lumen in the proximal vessel to the true lumen in the distal vessel via the subintimal space. The main obstacle in this procedure was delivering large enough balloons retrograde and then removing the winged balloon back through the fragile collaterals.

Since the initial descriptions of the retrograde approach and CART, there have been many advances in wire and catheter technologies. These advances, along with a co-operative effort by many high volume CTO operators, have allowed the retrograde approach to be more widely adopted and successful.

Wire technology has included the SION (Asahi Intecc, Nagoya, Japan), SION Blue (Asahi Intecc, Nagoya, Japan), RG3 (Asahi Intecc, Nagoya, Japan) and the R350 (Vascular Solutions, Minneapolis, MN, USA). The Corsair catheter (Asahi Intecc, Nagoya, Japan) has been the major advance in catheter technology. The Corsair allowed for the advent of the reverse CART and subsequent variations including the catheter assisted reverse CART and snaring used for externalization.

## WIRE TECHNOLOGY

Traditionally, the collateral channels would be crossed with either a Fielder FC (Asahi Intecc, Nagoya, Japan) or a Fielder XT (Asahi Intecc, Nagoya, Japan). The tip of the Fielder XT is 0.009" and can cause dissection. In a septal channel, this is rarely of clinical consequence but may remove the only wireable retrograde option. Epicardial channels have a higher likelihood of being the only source of collateral flow and dissection can lead to ischemia.

Development of the SION and SION Blue has made crossing collateral channels a less daunting task. The SION wire uses double core technology to improve tip control. The soft tip (0.7gm deflection weight) along with the Slip Coat^®^ technology allows navigation of more tortuous anatomy within the collateral channel. The SION Blue wire has a softer tip (0.5gm deflection weight) but provides better support for delivery of equipment as the channel is crossed. These wires have allowed access across previously uncrossable tortuous collateral channels.

Externalization wires have been developed as well. The RG3 guidewire is a 330cm wire that is 0.010" diameter with the Slip Coat^®^ technology over the first 170cm. This allows for more pushability when externalizing as well as being supportive to deliver gear in an antegrade direction after externalization is complete. The R350 is a 350cm guidewire with a 0.013" diameter that is also frequently used for externalization. Both wires provide additional length, which is key to having a working length over the antegrade arm without losing control of the wire from the retrograde side.

## CATHETER TECHNOLOGY

The Corsair catheter use in crossing collateral channels was first described in 2010(3). The Corsair is able to traverse tortuous anatomy and dilates the channel as it is advanced. Because of the design, the catheter is rotated with minimal forward pressure and allowed to slowly advance. As it advances, the catheter also dilates the channel. The dilation of the channel allows for two things to occur. First, it makes removal of the catheter easier and more predictable. Secondly, if necessary, other gear such as balloons can be exchanged over the retrograde wire. The Corsair is able to advance through collaterals that were previously unable to be crossed and can do so in an efficient manner.

Reverse CART has evolved as a more predictable approach to successful retrograde recanalization of CTO. In this procedure, the Corsair catheter retrograde and a balloon catheter antegrade are introduced into the subintimal space. A common subintimal space is created and the retrograde wire is advanced into the true lumen in the proximal vessel. Details of this procedure have been previously described [4]. 

Following wiring of the proximal true lumen, the wire is then externalized. The more common, but less predictable, technique is to pass the retrograde wire into the antegrade guide. The Corsair is advanced into the antegrade guide and the wire is then exchanged for a longer wire. Both the R350 and the RG3 are the preferred wires. This wire is then passed out the antegrade guide and can then be used with a monorail system to do balloon angioplasty and subsequent stenting. 

Using a wire snare via the antegrade guide to capture the retrograde wire is also used in some cases. In this situation, the retrograde wire is advanced into the aorta and an 18 mm by 30 mm EN Snare(Merit Medical Systems, South Jordan, UT, USA) is used to capture it. This is ideally done in the Sinus of Valsalva or at the mouth of the innominate artery. Once a wire is snared, it cannot be removed retrograde unless the bent portion is removed after externalization.

The catheter assisted reverse CART is another technique that has evolved in recent years [5]. In this variation, either a Guideliner (Vascular Solutions, Minneapolis, MN, USA) or a Guidezilla (Boston Scientific, Maple Grove, MN, USA) is advanced over the antegrade balloon into the subintimal space. This allows for two things to happen. First, when the antegrade balloon is deflated, it acts as a scaffold to prevent the space from collapsing. Secondly, once the retrograde wire is into the catheter, it provides a direct path to the guide and subsequent externalization. Because coronary arteries with CTOs tend to be diffusely diseased, it alleviates the need to wire complex lesions once in the proximal true lumen.

## TECHNIQUES

Selective injection into collateral channels is frequently used to visualize a path. Although this has a low complication rate of dissection or perforation, it may not be necessary. A technique known as septal surfing has been advocated for septal channels. Frequently, connections between the left anterior descending and posterior descending artery may not be visible, yet exist. If the septal channel can be crossed without selective injection, a potential complication can be avoided. If the septal channel cannot be crossed, a selective injection can be considered. Epicardial channels should never be surfed.

Using a knuckle wire to quickly cross long segments of occlusion has become a widely accepted technique. In this situation, a hydrophilic jacketed wire is made to prolapse. When in the subintimal space, this prolapsed wire will bluntly dissect the tissue plane. It is able to be advanced safely, including in tortuous and ambiguous anatomy. The knuckle wire will take the path of least resistance and will not perforate the adventitia. The knuckle wire can be used both antegrade and retrograde when performing reverse CART.

Due to excessive length and tortuosity, externalizing a wire can be difficult. Rotaglide^®^ lubricant is used to ease this process. After the retrograde wire and Corsair catheter are in the antegrade guide, the wire is removed and Rotaglide^®^ is flushed through the Corsair prior to introducing the externalization wire. This allows for easier passage of the externalization wire through the entire system.

## SUMMARY

The learning curve for the retrograde approach to coronary CTO remains steep. Initially, standard guidewires were used to cross collateral channels and balloon catheters then had to navigate the anatomy in order to attempt revascularization. Subsequently, wire technology with the SION and SION Blue guidewires has made it possible to get into the distal vascular bed in previous impossible anatomy. Catheter technology has provided the Corsair catheter allowing gear to be delivered over the retrograde wire through tortuous anatomy.

Finally, open dialogue and communication between high volume operators internationally has led to rapidly advancing techniques allowing for safer, more efficient and ultimately more successful procedures.

## CONFLICT OF INTEREST

The author confirms that this article content has no conflict of interest.
